# An eye-tracking study for measuring the attentional characteristics towards emotional scenes in children with autism spectrum condition

**DOI:** 10.1192/j.eurpsy.2021.605

**Published:** 2021-08-13

**Authors:** M. Lizarán, R. Sahuquillo-Leal, P. Navalón, A. Moreno-Giménez, B. Almansa, M. Vento, A. García-Blanco

**Affiliations:** Neonatal Research Group, The Medical Research Institute Hospital La Fe (IIS La Fe), Valencia, Spain

**Keywords:** autism spectrum condition, emotions, Eye movements, childhood

## Abstract

**Introduction:**

The difficulties in social interaction present in individuals with autism spectrum conditions may are related with the abnormal attentional processing of emotional information. Specifically, it has been hypothesized that the hypersensibility to threat shown by individuals with autism may explain an avoidance behaviour. However, this hypothesis is not supported by research and the underlying psychological mechanisms of social interaction in autism still unclear.

**Objectives:**

The aim of the present study was to examine attentional processing biases by administering a computer-based attentional task in a sample of 27 children with autism spectrum conditions and 25 typically developed participants (age 11-15 years).

**Methods:**

The initial orienting of attention, the attention al engagement, and the attentional maintenance to different emotional scenes in competition (i.e. happy, neutral, threatening and sad) were measured by recording the eye movements during a 20 seconds free-viewing task.

**Results:**

The main findings were: i) children with autism spectrum conditions showed an initial orientating bias towards threatening stimuli; and ii) while typically developed children revealed an attentional engagement and attentional maintenance bias towards threatening stimuli, children with autism spectrum conditions did not.

**Conclusions:**

The findings of the present study are consistent with the affective information processing theories and shed light on the underlying mechanisms of social disturbances in autism spectrum conditions.

